# Host genetic variation is associated with root-associated microbiome assembly and resistance to *Fusarium oxysporum* in tobacco

**DOI:** 10.1093/plphys/kiag727

**Published:** 2026-09-30

**Authors:** Huan Su, Huabing Liu, Jiemeng Tao, Zechao Qu, Qiao Wang, Peng Lu, Xinhua Tian, Shizhou Yu, Kaiyan Li, Heli Shi, Mei Zuo, Bikun Xiang, Jizhong Wu, Peijian Cao, Jingjing Jin

**Affiliations:** China Tobacco Gene Research Center, Zhengzhou Tobacco Research Institute of CNTC, Zhengzhou 450001, China; Beijing Life Science Academy, Beijing 102200, China; Technology Center, China Tobacco Anhui Industrial Co., Ltd, Hefei 230088, China; China Tobacco Zhejiang Industrial Co., Ltd, Hangzhou 310008, China; China Tobacco Gene Research Center, Zhengzhou Tobacco Research Institute of CNTC, Zhengzhou 450001, China; Beijing Life Science Academy, Beijing 102200, China; China Tobacco Gene Research Center, Zhengzhou Tobacco Research Institute of CNTC, Zhengzhou 450001, China; China Tobacco Gene Research Center, Zhengzhou Tobacco Research Institute of CNTC, Zhengzhou 450001, China; Beijing Life Science Academy, Beijing 102200, China; China Tobacco Gene Research Center, Zhengzhou Tobacco Research Institute of CNTC, Zhengzhou 450001, China; Beijing Life Science Academy, Beijing 102200, China; China Tobacco Gene Research Center, Zhengzhou Tobacco Research Institute of CNTC, Zhengzhou 450001, China; Beijing Life Science Academy, Beijing 102200, China; Guizhou Academy of Tobacco Science, Guiyang 550081, China; China Tobacco Zhejiang Industrial Co., Ltd, Hangzhou 310008, China; Enshi Tobacco Company of Hubei Province, Enshi, Hubei 445000, China; Enshi Tobacco Company of Hubei Province, Enshi, Hubei 445000, China; Enshi Tobacco Company of Hubei Province, Enshi, Hubei 445000, China; China Tobacco Zhejiang Industrial Co., Ltd, Hangzhou 310008, China; China Tobacco Gene Research Center, Zhengzhou Tobacco Research Institute of CNTC, Zhengzhou 450001, China; Beijing Life Science Academy, Beijing 102200, China; Key Laboratory of Biosynthesis and Biomanufacturing in Model Plants (Beijing Life Science Academy), Ministry of Industry and Information Technology, Beijing 102200, China; China Tobacco Gene Research Center, Zhengzhou Tobacco Research Institute of CNTC, Zhengzhou 450001, China; Beijing Life Science Academy, Beijing 102200, China

## Abstract

Plant–microbe interactions are essential for plant health, yet the genetic basis of host control over root-associated microbiome assembly in *Nicotiana tabacum* remains unclear. We integrated whole-genome resequencing, amplicon sequencing, and metabolomics across 50 tobacco cultivars, together with microbial genome-wide association and mediation analyses, to investigate host–microbiome covariation. Host genotype significantly influenced the diversity and composition of root-associated microbial communities, particularly in the rhizosphere. Several host loci were associated with heritable microbial taxa linked to nutrient acquisition and stress responses. Mediation analysis suggested that associations between host genetic variation and *Pseudomonadaceae* abundance were partly transmitted through secondary metabolite variation, particularly terpenoids, whereas this mediating effect was less pronounced for other taxa. Functional assays further showed that several genotype-associated bacterial isolates antagonized *Fusarium oxysporum*. Among them, *Pseudomonas aeruginosa* FZ1 suppressed pathogen growth, enhanced host defense responses, and reduced disease severity in greenhouse experiments. These findings suggest that host genetic variation is associated with shifts in the tobacco root microbiome and that secondary metabolites may contribute to the assembly of specific microbial taxa. The study provides a basis for future mechanistic and breeding-oriented investigations of microbiome-associated traits.

## Introduction

Biodiversity conservation is a fundamental basis for sustainable agricultural development, and the plant microbiome represents a core component of agricultural biodiversity, playing indispensable roles in crop health, productivity, and stress resilience (Müller et al. 2016; Hegazi et al. 2019; Qiu et al. 2019). Diverse symbiotic microorganisms, including bacteria, fungi, and viruses, colonize different plant tissues and contribute to mineral nutrient acquisition, disease resistance, abiotic stress tolerance, and the regulation of plant growth and development (Trivedi et al. 2020; Bai et al. 2022). Although the ecological and functional importance of plant-associated microbiomes is widely recognized, only a small fraction of plant–microbe interactions has been functionally characterized, and the regulatory mechanisms governing microbiome assembly remain incompletely understood.

The composition and function of plant microbiomes are shaped by multiple factors, including soil properties (Buckley and Schmidt 2003), plant compartments (YANG et al. 2023), developmental stage (Xiong et al. 2021; Olimi et al. 2022), environmental conditions (Trivedi et al. 2020), plant immune status (Wang et al. 2022a), and host genotype (Zhang et al. 2023). Among these, host genotype has emerged as a critical intrinsic determinant of microbiome variation. Increasing evidence indicates that host genetic effects on microbiome assembly are not uniform but operate through distinct regulatory architectures. Two non-mutually exclusive regulatory modes have been proposed. The first, metabolite-mediated regulation, posits that genetically controlled metabolites act as the primary ecological filters shaping microbial colonization (Su et al. 2025). The second, genotype-integrated regulation, posits that host genetic variation influences microbial communities through additional mechanisms beyond quantifiable metabolites alone, including root architecture, immune signaling, cell wall properties, and temporal patterns of exudation (Chen et al. 2020; He et al. 2024). Discriminating between these regulatory modes is essential for understanding how host genetic variation shapes the rhizosphere microbiome.

Host genetic regulation of the microbiome has been reported across multiple crop species. In rice, specific genetic loci were shown to influence the abundance of beneficial taxa such as *Burkholderia* and *Pseudomonas*, contributing to improved nutrient utilization and stress tolerance (Zhang et al. 2019; Su et al. 2024). In maize, host genetic variation was associated with changes in microbial community composition and root exudate profiles, affecting root development and abiotic stress adaptation (Wallace et al. 2018; He et al. 2024). Similar genotype-dependent microbiome patterns have been observed in tomato and other crops (Deng et al. 2021; Wang et al. 2022b; Edwards et al. 2023; Xun et al. 2024). Although environmental factors, particularly soil properties, often explain the majority of microbiome variation, the genetically structured fraction, even if quantitatively modest, can exert disproportionate effects on plant performance by selectively recruiting functionally important microbial partners. This genetically controlled microbiome fraction therefore represents a highly promising target for microbiome-informed breeding strategies (Xun et al. 2024).

The genetically structured microbiome fraction is of particular relevance to disease resistance. Plant-associated microbiomes, especially those in the rhizosphere, constitute a critical first line of defense against soil-borne pathogens through microbial competition, antibiosis, and niche exclusion (Trivedi et al. 2020; Pereira et al. 2023). Among soil-borne pathogens, *Fusarium oxysporum* is an important pathogen of diverse crops, owing to its broad host range, high genetic diversity, and ability to persist in soil as chlamydospores, which can complicate disease management (Gai et al. 2021; Xie et al. 2025). The susceptibility of *F. oxysporum* to microbially produced antifungal compounds further makes it an ecologically relevant model for studying microbiome-associated disease suppression (Tintor et al. 2020; Song et al. 2026). However, the extent to which plants can genetically program the recruitment of disease-suppressive microbiota remains poorly understood. Tobacco (*Nicotiana tabacum* L.) and related *Solanaceae* species provide a particularly well-suited system for investigating host genetic control over microbiome assembly. Their root exudates are rich in alkaloids (eg nicotine, anabasine), terpenoids, and phenylpropanoids, many of which possess antimicrobial or signaling activities that selectively shape rhizosphere microbial assemblages (Fiesel et al. 2022). The genetic regulation of these specialized metabolic pathways varies substantially among tobacco cultivars, creating a natural framework in which host metabolic diversity may translate into differential microbial recruitment. This genetically tractable metabolic variation, combined with the diversity of commercial tobacco germplasm spanning distinct genetic lineages and cultivation backgrounds, makes tobacco an excellent model for dissecting genotype-driven microbiome assembly and its consequences for disease resistance.

Recent advances in high-throughput sequencing and multi-omics technologies have greatly expanded the scope of plant microbiome research. Genome-wide association studies (GWAS) provide a powerful framework to link host genetic variation with complex traits, including microbiome composition and function. Host functional genes can influence root-associated microbial communities through multiple mechanisms, such as altering root morphology, nutrient uptake efficiency, immune responses, and the secretion of secondary metabolites and phytohormones, thereby reshaping the root-associated microenvironment or directly affecting microbial behavior (Liu et al. 2023). Importantly, integrative GWAS approaches that jointly consider host genotype, metabolite variation, and microbial traits enable the dissection of metabolite-mediated versus genotype-integrated modes of microbiome regulation. Previous microbial GWAS (MGWAS) studies in crops such as rice, maize, tomato, and tea have demonstrated that specific host loci can selectively enrich beneficial microbial taxa, ultimately influencing nutrient use efficiency, stress tolerance, and yield-related traits (Zhang et al. 2019; Oyserman et al. 2022; Tan et al. 2022; He et al. 2024). These findings support the concept that the plant microbiome can be viewed as an extension of the host phenotype shaped by genetic variation.

Despite these advances, significant challenges remain in translating plant microbiome knowledge into agricultural applications. The mechanistic links connecting host genetic variation, metabolic traits, and microbiome assembly remain poorly understood, and the ecological stability and functional reliability of beneficial microorganisms across genetically diverse tobacco cultivars require further evaluation. Here, we investigated the genetic basis of root-associated microbiome assembly and its functional consequences in cultivated tobacco. By integrating whole-genome resequencing, amplicon sequencing, metabolomics, and transcriptomics across 50 diverse tobacco cultivars, we characterized host genetic determinants of microbial recruitment, evaluated the contribution of secondary metabolites to host–microbiome interactions, and identified genotype-associated microbial taxa linked to disease-suppressive potential. Combining microbial genome-wide association analyses, mediation modeling, and functional validation of representative isolates, we further assessed the capacity of recruited microorganisms to suppress the soil-borne pathogen *F. oxysporum*. Together, this study provides a systems-level framework for understanding host genetic control of plant microbiomes and offers insights into microbiome-assisted strategies for crop improvement and disease management.

## Materials and methods

### Experimental design

The field experiment was conducted at Yuanyang County, Xinxiang City, Henan Province (113.73° E, 35.02° N), at a site with uniform soil conditions and well-established irrigation and drainage systems. The soil was classified as sandy soil, with a pH of 8.12 ± 0.17, available nitrogen of 55.73 ± 6.25 mg/kg, available phosphorus of 19.71 ± 3.87 mg/kg, and available potassium of 214.19 ± 45.57 mg/kg. Soil physicochemical properties were determined according to standard analytical procedures (Cheng et al. 2024). A total of 50 representative tobacco cultivars encompassing diverse genetic backgrounds, including flue-cured, burley, oriental, sun-cured, cigar, and rustica, were selected for analysis (Table S1). The experiment was established in July 2023. Each cultivar was planted in an independent plot consisting of 3 rows with 8 plants per row (24 plants per plot). The positions of the cultivar plots were spatially randomized across the field to minimize potential spatial and environmental effects. For each cultivar, 8 individual plants were independently sampled and processed as biological replicates for subsequent sequencing and analyses. These biological replicates were derived from the same cultivar plot and therefore did not represent independent field or plot replicates. Tobacco plants were grown on ridges 32 to 36 cm in height, with a plant spacing of 45 to 48 cm and a row spacing of 1.25 m.

### Root-associated sample collection, DNA extraction, and bioinformatic analysis

For each cultivar, 4 distinct root-associated ecological niches were sampled following established protocols (Cheng et al. 2024): bulk soil (BS), rhizosphere soil (RS), rhizoplane (RP), and root endosphere (RE). Eight independent plants per cultivar (one plant per plot within each block; see Experimental Design) were destructively sampled as true biological replicates. Each replicate sample represented a single plant, and all analyses treated individual plants as the experimental unit. Specifically, after uprooting the tobacco plants, approximately 200 g of soil from the root pit was sieved to remove large impurities and homogenized as the BS sample. The roots were then gently shaken to discard loosely adhering soil; the soil tightly adhering to the root surface (∼50 g) was collected using a soft brush to represent the RS. Both BS and RS samples were immediately wrapped in aluminum foil and cryopreserved in liquid nitrogen. To isolate the RP fraction, 5 g of root tissue was submerged in 15 mL of sterile phosphate-buffered saline, followed by sonication and centrifugation. For the RE fraction, the remaining roots were surface-sterilized by sequential immersion in 75% ethanol (5 min), 1% sodium hypochlorite (5 min), and 75% ethanol (30 s), followed by 3 rinses with sterile water. The sterilized tissues were subsequently pulverized in liquid nitrogen for total genomic DNA extraction.

Total genomic DNA was extracted from all samples according to our previously described protocol (Tao et al. 2022). A total of 1,600 samples (50 genotypes × 4 niches × 8 replicates) were subjected to amplicon sequencing. Bacterial 16S rRNA V5-V7 regions were amplified using 799F/1193R primers, while fungal ITS1 regions were targeted with ITS1F/ITS2 primers (Table S2). Sequencing libraries were constructed and sequenced on the Illumina NovaSeq 6000 platform (Cheng et al. 2023, 2024). Raw sequencing data were processed for quality control via fastp (v0.23.1) (Chen 2023), and paired-end reads were merged using FLASH (v1.2.11) (Magoc and Salzberg 2011). After primer removal with Cutadapt (v5.0) (Martin 2011) and chimera filtering, amplicon sequence variants (ASVs) were generated using the DADA2 module within QIIME2 (v2023.2) (Callahan et al. 2016; Estaki et al. 2020). Taxonomic annotation was performed against the SILVA 138 prokaryotic and UNITE 9 eukaryotic databases, with sequences assigned to chloroplasts and mitochondria subsequently excluded. Microbial community analysis, including diversity metrics, was conducted using the phyloseq R package (McMurdie and Holmes 2013). PERMANOVA was employed to evaluate the relative contributions of environment, genotype, and tobacco class to community variance (Oksanen et al. 2012). Finally, functional potentials were predicted using PICRUSt2 (v2.5.2) for bacteria (Douglas et al. 2020) and FUNGuild (v1.1) for fungi (Nguyen et al. 2016).

### Tobacco leaf sample collection, DNA extraction, and genomic analysis

Leaf samples were harvested from the same cohort of tobacco plants used for soil sampling. For each cultivar, healthy individuals exhibiting uniform growth and no visible disease symptoms were selected. Approximately 20 g of middle leaves were harvested, immediately wrapped in aluminum foil, flash-frozen in liquid nitrogen, and transported on dry ice. All samples were stored at −80 °C until DNA extraction. Genomic DNA was fragmented to an average size of 350 bp via ultrasonication, and sequencing libraries were constructed and sequenced by Novogene Biotechnology Co., Ltd (Beijing, China).

Raw sequencing data were preprocessed for quality control using fastp (v0.23.1) (Chen 2023). The cleaned reads were aligned to the *N. tabacum* cv. “Petite Havana SR1” reference genome (Wang et al. 2024) using BWA-MEM 2 (v2.2.1) (Vasimuddin et al. 2019). The resulting SAM files were converted, sorted, and stripped of PCR duplicates using SAMtools (v1.17) (Danecek et al. 2021) and sambamba (v1.0.0) (Tarasov et al. 2015). For robust variant discovery, SNP calling was performed using a consensus approach with bcftools (v1.17) (Danecek et al. 2021), freebayes (v1.3.6) (Garrison and Marth 2012), and gatk4 (v4.4.0) (McKenna et al. 2010), from which common biallelic SNP sites were extracted. These variants were further refined using VCFtools (v0.1.16) (Danecek et al. 2011) based on stringent criteria: a missing rate < 0.1, a minor allele frequency > 0.05, and a Hardy–Weinberg equilibrium *P*-value > 0.0001. High-quality SNPs were then imputed using Beagle (v5.4) (Browning et al. 2021) with default parameters. Functionally annotated was performed using snpEff (v4.5) (Cingolani et al. 2012) with default parameters based on the corresponding genome annotation. Population genetic structure was characterized using ADMIXTURE (v1.3.0) (Alexander et al. 2009) after LD pruning with PLINK2 (--indep-pairwise 500 100 0.2) (Chang et al. 2015). ADMIXTURE was run with *K* = 1 to 10 and one run per *K* using cross-validation.

### Rhizosphere metabolite profiling and analysis

Rhizosphere soil samples were vacuum freeze-dried and extracted with 70% aqueous methanol (v/v) containing internal standards at a solid-to-liquid ratio of 1:30 (w/v). Samples were vortexed, incubated in a water bath to facilitate metabolite extraction, and centrifuged to collect the supernatant. The extracts were subsequently filtered through a 0.22 *μ*m membrane and transferred to autosampler vials for LC–MS/MS analysis. The sample extracts were analyzed using an UPLC-ESI-MS/MS system consisting of an ExionLC AD UPLC system and a tandem mass spectrometry system (SCIEX, China).

Metabolite separation was performed on an Agilent SB-C18 column (1.8 *μ*m, 2.1 mm × 100 mm). The mobile phases consisted of ultra-pure water containing 0.1% formic acid (A) and acetonitrile containing 0.1% formic acid (B). The elution gradient was programmed as follows: 0 to 9 min, 5% to 95% B; 9 to 10 min, 95% B; 10 to 11 min, 95% to 5% B; and 11 to 14 min, 5% B. The flow rate was maintained at 0.35 mL min^−1^, the column temperature at 40 °C, and the injection volume at 2 *μ*L.

Mass spectrometric detection was performed using an electrospray ionization (ESI) source coupled to a triple quadrupole mass spectrometer operating in multiple reaction monitoring mode. The ion source temperature was set to 500 °C, with spray voltages of 5,500 and −4,500 V in positive and negative ion modes, respectively. Ion source gas I, gas II, and curtain gas were maintained at 50, 60, and 25 psi, respectively. Declustering potential and collision energy were optimized individually for each metabolite. Metabolites were identified by comparison of MS/MS fragmentation patterns with reference standards or public spectral databases, and detailed metabolite information is provided in Table S3.

Differential accumulation of metabolites was determined using the limma R package (Ritchie et al. 2015). Furthermore, modular analysis of metabolites was performed using the WGCNA (weighted gene co-expression network analysis) package (Langfelder and Horvath 2008). Partial least squares discriminant analysis (PLS-DA) was performed using the ropls R package (Thévenot et al. 2015). Variable Importance in Projection scores > 1.0 were considered indicative of metabolites contributing significantly to group separation.

### Tobacco root transcriptome sequencing and analysis

The transcriptome sequencing for tobacco roots was performed by Novogene Biotechnology Co., Ltd (Tianjin, China). Root tissues were collected at 7 d postinoculation from 3 treatments: the sterile-water control (CK), *F. oxysporum* alone (Fo), and *F. oxysporum* + FZ1 (Fo + FZ1). Approximately 1 g of root tissue was collected from each biological replicate, gently washed with running water to remove soil, immediately frozen in liquid nitrogen, and stored at −80 °C until RNA extraction. Three biological replicates were included for each treatment, and each biological replicate was processed independently and used to construct an individual sequencing library, resulting in 9 libraries in total.

Raw sequencing data were subjected to stringent quality control using fastp (0.23.1). Salmon (v1.10.1) (Patro et al. 2017) was used for quantification of clean reads. The R package tximport (v1.26.1) (Soneson et al. 2015) was used to calculate TPM (Transcripts Per Kilobase Million) values, and differential expression analysis was performed using the edgeR method implemented in the R package RNAseqStat2 (v0.1.0) (Zeng and Xia 2022), with Fo vs CK and Fo + FZ1 vs CK. Genes with *P* < 0.05 and an absolute log_2_ fold change (|log_2_FC|) ≥ 2.5 were considered differentially expressed.

### High-throughput microbial isolation and culture

To maximize the recovery of cultivable microorganisms and establish a representative culture collection, root-associated microbial samples from all tobacco cultivars were pooled prior to microbial isolation. Bacterial isolation and culture were performed according to the method described by Zhang et al. (2021) and combined with the previous optimization protocol from our laboratory (Cheng et al. 2023, 2024). Briefly, liquid medium limited dilution was used to disperse individual bacteria into a 96-well culture plate. High-throughput bacterial isolation and identification were conducted by amplifying the V5 to V7 regions of the 16S rRNA gene and adding barcodes for sequencing. Fungi were isolated using the plate dilution method on potato dextrose agar (PDA) medium supplemented with streptomycin, and pure cultures were obtained through continuous streaking. DNA extraction was performed using the E.Z.N.A. Fungal DNA Kit (Omega Biotek Inc., Doraville, GA, USA). The ITS1F and ITS2 primers were used to amplify the ITS1 region. After Sanger sequencing, the sequences were compared to the UNITE 9 database (Nilsson et al. 2019) for identification, and the strains were preserved in glycerol at −80 °C. The ASV sequences were aligned using MAFFT (v7.520) (Katoh et al. 2002), trimmed using trimAL (v1.4.rev15) (Capella-Gutierrez et al. 2009), and a phylogenetic tree was constructed based on Maximum Likelihood (ML) using IQ-TREE 2 (v2.2.2.6) (Minh et al. 2020).

### Bacterial whole-genome sequencing and assembly

To obtain high-quality genomic DNA, single bacterial colonies were inoculated into 1.5 L LB medium in 3 L conical flasks and cultured in a shaking incubator (28 °C, 200 rpm). Upon reaching an OD600 of 0.5 to 1.0, cells were harvested by centrifugation at 4,000 rpm for 20 min at 4 °C. The resulting bacterial pellets were washed 3 to 5 times with sterile water to eliminate residual medium, flash-frozen in liquid nitrogen, and stored at −80 °C.

Whole-genome sequencing was conducted by Novogene Biotechnology Co., Ltd (Beijing, China) using PacBio and Oxford Nanopore long-read sequencing technologies. Quality control of sequencing data was performed using fastp (0.23.1) (Chen 2023). De novo assembly was executed using SOAPdenovo2 (Luo et al. 2012), SPAdes (v4.0.0) (Bankevich et al. 2012), and ABySS (v2.3.8) (Simpson et al. 2009), with the resulting contigs integrated via CISA (Lin and Liao 2013) to generate a high-quality consensus assembly. Gaps in the initial scaffolds were further refined and closed using GapCloser (Xu et al. 2020). Functional annotation of the assembled genomes was performed against multiple comprehensive databases, including GO, KEGG, NCBI nonredundant, SwissProt (Bairoch and Apweiler 2000), clusters of orthologous groups (Galperin et al. 2025), and transporter classification database (Saier et al. 2021). Furthermore, the antiSMASH 7.0 (Blin et al. 2023) database was employed to identify and characterize biosynthetic gene clusters (BGCs) involved in secondary metabolite production.

### Microbiome and genotype association analysis

Broad-sense heritability (*H*^2^) of the microbiota was estimated at the family, genus, and ASV levels using the sommer R package (Covarrubias-Pazaran 2016), with each ecological niche analyzed independently. To investigate the influence of host genetic variation on root microbiome composition, MGWAS was conducted using microbial abundance as phenotypes. Microbial abundances were first normalized using cumulative sum scaling and subsequently subjected to a rank-based inverse normal transformation. The distribution of transformed traits was assessed using the Shapiro–Wilk test implemented in R, confirming their suitability for downstream linear mixed models (LMMs) analyses. Best linear unbiased predictions were then calculated to account for environmental and batch effects. MGWAS was conducted using GEMMA software (v0.98) (Zhou and Stephens 2012). The first 10 principal components derived from host genomic variation were included as fixed effects to control for population structure, and a kinship matrix calculated from genome-wide SNP data was incorporated as a random effect. Model performance and potential systematic biases were evaluated using quantile–quantile (Q–Q) plots. Multiple testing correction was performed using the Bonferroni method, with a significance threshold of 3.17 × 10^−7^ (0.05/157,820 SNPs). For candidate gene identification, significant SNPs were analyzed separately for bacterial and fungal traits. Each significant SNP was assigned a 100-kb window centered on the SNP (±50 kb), and overlapping windows were merged into nonredundant loci. Each resulting locus was then extended by 50 kb on both sides to define the candidate genomic region and capture potential regulatory elements.

To further evaluate whether metabolite modules may mediate the associations between host genetic variation and microbial abundance, we performed mediation analysis using 3 linked LMMs implemented in the lmerTest R package (Kuznetsova et al. 2017). The first model evaluated the association between host genotype and metabolite module (path *a*). The second evaluated the association between metabolite module and microbial abundance (path *b*). A joint model (BLUP ∼ module + SNP + (1|TaxID)) was used to estimate the direct effect (*c′*). The indirect effect (*a* × *b*) and proportion mediated [*a* × *b*/(*a* × *b* + *c′*)] were then estimated. The significance of the indirect effect was assessed using the Sobel test and 1,000-resample bootstrap confidence intervals. An indirect effect was considered significant when its 95% bootstrap confidence interval did not include zero. Effect sizes and confidence intervals for all tested mediation pathways were provided in Table S4.

### In vitro antagonistic activity assay

The tobacco root rot pathogen, *F. oxysporum*, was provided by the Yunnan Academy of Tobacco Agricultural Sciences. The antagonistic potential of bacterial isolates was evaluated using the dual-culture plate confrontation method. Briefly, a plug of actively growing *F. oxysporum* mycelium was inoculated into the center of a PDA plate. The candidate bacteria were cultured in LB liquid medium at 28 °C until reaching an OD_600_ of approximately 0.5. Subsequently, 5 *μ*L of the bacterial suspension was inoculated at designated positions 2.5 cm from the central pathogen plug. PDA plates inoculated with the pathogen alone served as controls. All plates were incubated in an inverted position at 28 °C. Antagonistic effects were assessed by monitoring the inhibition zones once the pathogen in the control plates reached full confluence.

### Assessment of plant growth-promoting traits

To evaluate the plant growth-promoting potential of the isolates, we assessed their abilities in phosphate solubilization (using both inorganic and organic phosphorus media), potassium release (Alexandrov medium), siderophore production (Chrome Azurol S [CAS] medium), and nitrogen fixation (Ashby nitrogen-free medium). All media were purchased from Qingdao Hope Bio-Technology Co., Ltd (Qingdao, China). The standard 4-quadrant streak method was used for inoculation, and incubation conditions were the same as described above.

### Plant inoculation and biocontrol assay

To evaluate the biocontrol efficacy under greenhouse conditions, *F. oxysporum* was cultured to obtain a conidial suspension for plant inoculation. Briefly, 5 agar plugs were excised from the actively growing margin of an *F. oxysporum* colony and inoculated into potato dextrose broth supplemented with chloramphenicol (500 mL medium in a 1,000 mL Erlenmeyer flask). Cultures were incubated at 28 °C with shaking at 200 rpm for 36 h to induce sporulation. The culture was filtered through 2 layers of sterile gauze to remove mycelial fragments, and the filtrate was centrifuged at 8,000 rpm for 20 min. The resulting conidial pellet was resuspended in sterile distilled water, and spore concentration was adjusted to 1 × 10^6^ conidia mL^−1^ using a hemocytometer.

The inoculation experiment was conducted using the tobacco cultivar Zhongyan100, which was selected because it represents the genetic diversity of the association population and exhibited moderate susceptibility to *F. oxysporum* in preliminary pathogenicity assays. Four-week-old healthy seedlings were pretreated with 10 mL of antagonistic bacterial suspension (OD_600_ ≈ 0.5) 24 h before pathogen inoculation. As a bacterial control, *Escherichia coli*, a non-rhizosphere-associated and nonantagonistic strain, was cultured under identical conditions and applied at the same cell density. For pathogen challenge, approximately one-third of the root tips were trimmed prior to replanting, and each seedling was inoculated with 10 mL of *F. oxysporum* conidial suspension (1 × 10^6^ conidia mL^−1^) applied directly to the root zone. A pathogen-only treatment and a sterile-water control were included as positive and negative controls, respectively. Each treatment consisted of 10 biological replicates. Following inoculation, plants were maintained at 28 °C under a 16 h light/8 h dark photoperiod, and disease phenotypes were evaluated 7 d postinoculation.

### Statistical analysis

All statistical analyses were conducted in R (version 4.4.3) unless otherwise specified. One-way ANOVA followed by *Duncan*'s multiple range test was used for group comparisons. *Spearman* correlation analysis was applied to assess associations between microbial taxa and predicted functions. GO and KEGG enrichment analyses were performed using the clusterProfiler package, with Benjamini–Hochberg correction applied for multiple testing (False Discovery Rate < 0.05) (Wu et al. 2021).

## Results

### Host genotype affected tobacco root-associated microbial community structure and diversity

To explore the impact of different genotypes on the structure of tobacco root-associated microbial communities, amplicon sequencing was performed across multiple root-associated niches in 50 tobacco cultivars representing diverse genetic backgrounds. Microbial richness and evenness were assessed using the Observed and Shannon indices. The results revealed a decreasing trend in bacterial community diversity from the root distal end toward the root interior, with diversity in the RE being significantly lower than that in the other 3 ecological niches (Fig. 1a). A similar trend could also be observed for the fungal diversity (Fig. 1b).

**Figure 1:**
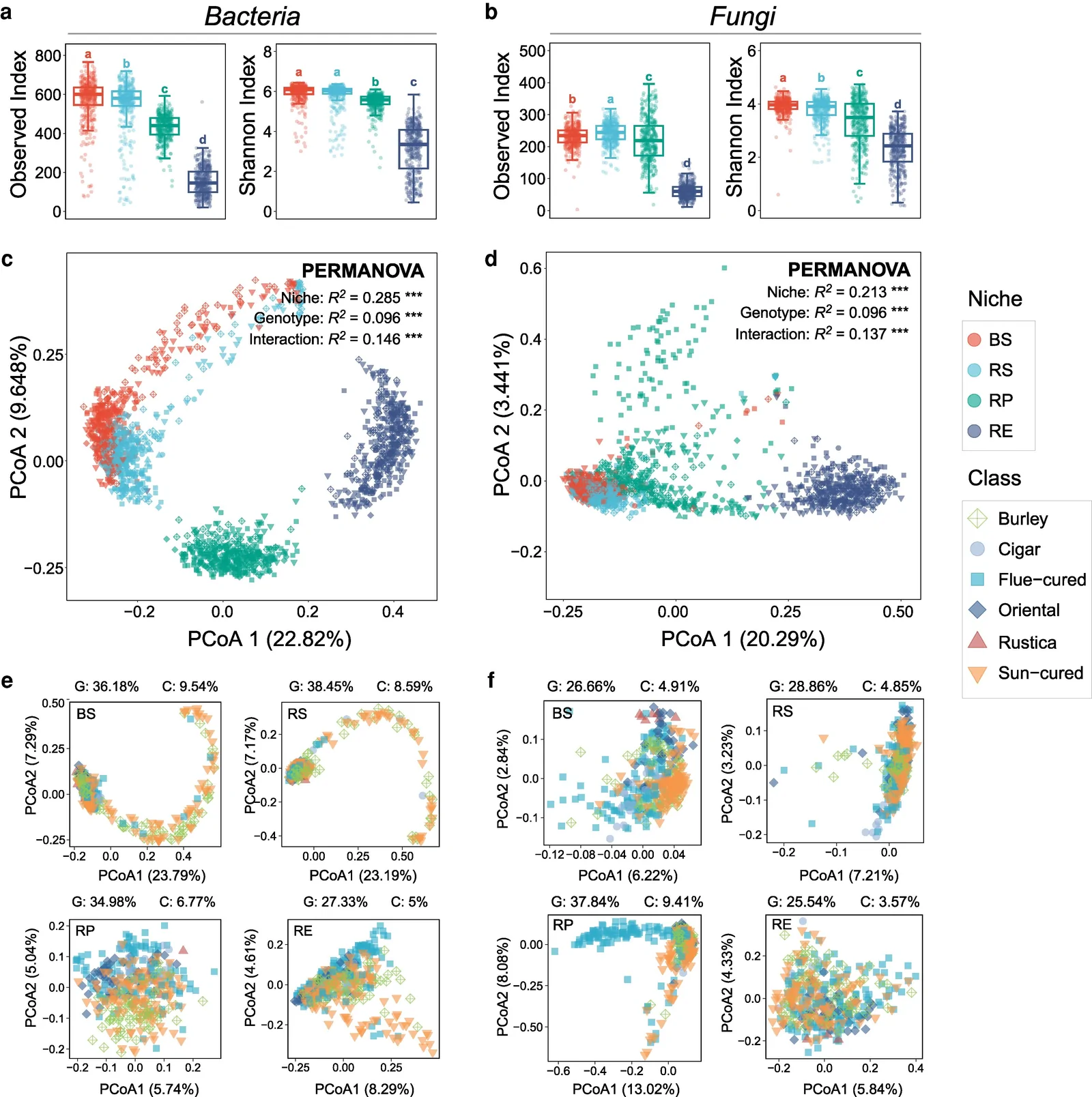
Diversity analysis of root-associated microbial communities in the tobacco population. a, b) α-Diversity of bacterial (a) and fungal (b) communities across 4 root-associated niches: bulk soil (BS), rhizosphere soil (RS), rhizoplane (RP), and root endosphere (RE) (*n* = 400 per niche). Different lowercase letters indicate significant differences among groups (*Duncan*'s multiple range test, *P* < 0.05). c, d) Principal coordinate analysis (PCoA) based on Bray–Curtis distances showing the distribution of bacterial (c) and fungal (d) communities across the 4 niches. e, f) PCoA of bacterial (e) and fungal (f) communities within each niche. The effects of niche, genotype (“G”), and tobacco curing class (“C”) on microbial community variation were assessed using PERMANOVA.

Principal coordinates analysis (PCoA) based on Bray–Curtis distance revealed significant differences in the bacterial (Fig. 1c) and fungal (Fig. 1d) community structures between different ecological niches (*P* < 0.01). PCoA analysis for bacterial and fungal communities exhibited similar patterns, with the RE differing significantly from the other 3 soil-related ecological niches (BS, RS, RP), while the differences between BS and RS samples were relatively small. Further PERMANOVA analysis indicated that the ecological niche had a large effect on microbial community structure (bacteria: *R*^2^ = 0.285, *P* < 0.001; fungi: *R*^2^ = 0.213, *P* < 0.001), while host genotype accounted for 9.6% of the variation in both bacterial and fungal community composition (*P* < 0.001), suggesting that genotype also had a significant impact on microbial communities.

Given the substantial effect of ecological niche on microbial communities, PCoA analysis for each individual ecological niche was performed to specifically evaluate the effect of genotype on microbial communities. The results revealed that genotype explained a substantial portion of the variability in bacterial and fungal communities across different ecological niches. Genotype explained 38.45% variance in rhizosphere bacteria and 37.84% in rhizoplane fungi (Fig. 1e, f). The effect of tobacco curing class on microbial communities was relatively small (3.57% to 9.54%). In the fungal community at the root surface, samples from flue-cured tobacco were distinctly different from the other samples, while the bacterial communities of burley and sun-cured tobacco showed certain similarities, suggesting that variations in rhizosphere microbial communities may be associated with differences in tobacco cultivation practices.

### Host genotype affected tobacco root-associated microbial community composition

To investigate the impact of tobacco genotype on the species composition of the root-associated microbial community, a systematic analysis of microbial communities for different ecological niches was conducted. The results showed that the microbial composition at the phylum level was generally consistent across the ecological niches, but significant differences in relative abundance were observed. The bacterial community was primarily composed of *Proteobacteria*, *Actinobacteriota*, *Acidobacteriota*, *Bacteroidota*, *Gemmatimonadota*, and *Firmicutes* (Fig. 2a), while the fungal community was dominated by *Ascomycota*, *Basidiomycota*, *Mortierellomycota*, *Chytridiomycota*, and *Glomeromycota* (Fig. 2b). The relative abundance of *Actinobacteriota* was significantly higher in root endosphere than in the other 3 ecological niches, suggesting their strong colonization advantage in the root interior environment (Fig. 2a). In the fungal community, *Glomeromycota* was highly enriched and almost exclusively detected in root endosphere samples, indicating that these fungi might form closer symbiotic relationships with the host (Fig. 2b). Additionally, microbial community composition appeared to show greater variation among cultivars in the endosphere than in the other root-associated niches (Fig. 2), suggesting that host genotype may contribute to differences in microbial colonization.

**Figure 2:**
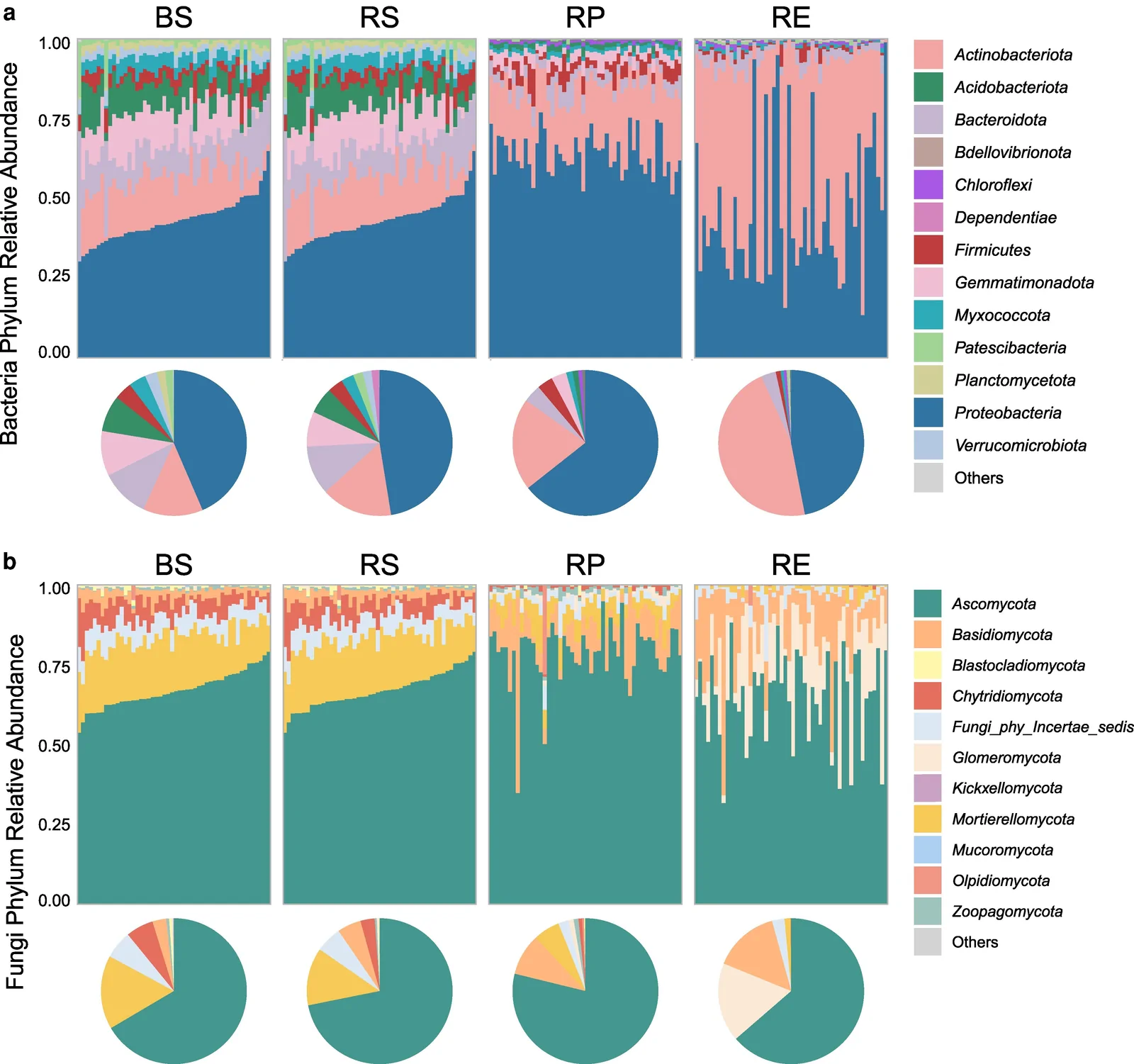
Taxonomic composition of root-associated microbial communities in the tobacco population. Relative abundance of bacterial (a) and fungal (b) phyla across tobacco cultivars and ecological niches (top), with a consistent cultivar order across niches. Pie charts (bottom) summarize the overall phylum-level composition within each ecological niche.

### Host genetics determines functionally heritable root-associated microbes

To explore the genetic basis of the root-associated microbial community driven by tobacco genotype, whole-genome resequencing on 50 tobacco cultivars was performed. A high-quality population genetic variation map containing 157,820 SNPs was constructed (Fig. S1a). These SNPs were relatively evenly distributed across the physical length of all chromosomes, with variants located in intergenic regions accounting for the largest proportion (79.20%) (Fig. S1b–d). Population structure and evolutionary analyses consistently separated the cultivars into 3 major genetic groups. The cultivars also exhibited substantial variation in quantitative traits, indicating considerable phenotypic diversity within the population (Fig. S1e, f and Table S5).

Based on this genome-wide variation map, we quantified the broad-sense heritability of root-associated microbial communities across distinct ecological niches at the genus, family, and ASV levels, aiming to evaluate the contribution of host genotype to microbial community assembly (Table S6). Notably, heritability estimates at the ASV level were significantly lower than those observed at the genus and family levels, suggesting that higher taxonomic ranks may integrate functionally or genetically coordinated microbial lineages, thereby enhancing the detectability of host genetic effects (Fig. 3a). Across all taxonomic resolutions, bacterial communities exhibited consistently higher heritability than fungal communities (Fig. 3b). Moreover, microbes inhabiting the root endosphere showed significantly lower heritability compared with those in soil-associated niches (Fig. 3a), implying that endosphere colonization was likely subject to stronger host filtering and environmental constraints that attenuated observable genetic effects. Further comparisons at the family level revealed heritability patterns highly consistent with those observed at the kingdom level: root endosphere microbes displayed the lowest heritability, while clear differentiation was evident among the 3 root-associated external niches. Collectively, these results indicate that host genetic effects are more pronounced in root-adjacent ecological compartments. Specifically, microbial groups such as *Gemmatimonadaceae*, *Micrococcaceae*, *Xanthomonadaceae*, and *Sphingomonadaceae* exhibited relatively high heritability (Fig. 3b). These taxa have been widely reported to participate in key plant-associated functions, such as nutrient acquisition, signal perception, and stress resistance, supporting the notion that host genetic variation is preferentially associated with functionally important members of the root-associated microbiome (Trivedi et al. 2020).

**Figure 3:**
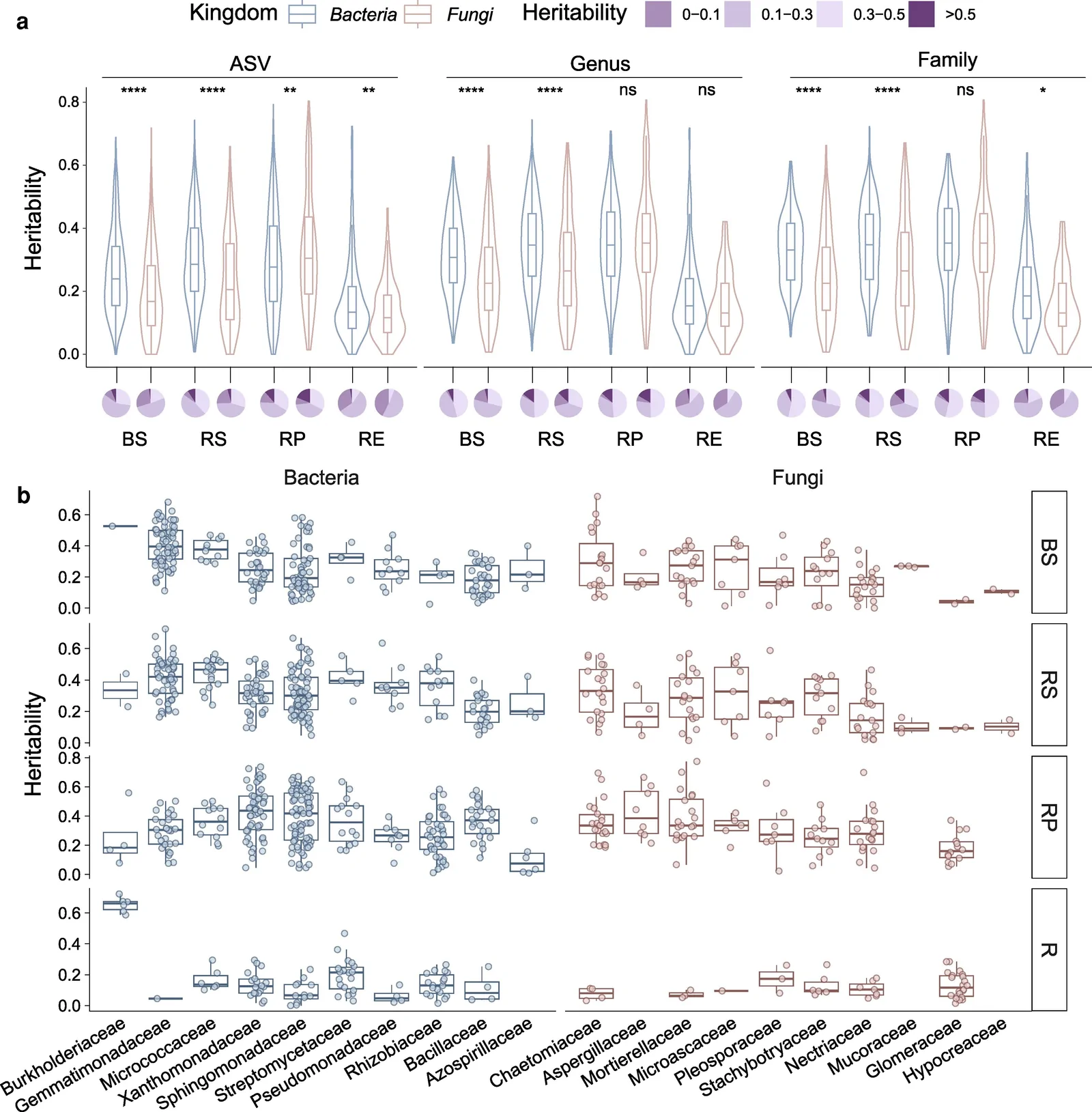
Broad-sense heritability analysis of the tobacco root-associated microbiome. a) Comparison of heritability estimates across taxonomic levels (family, genus, and ASV), with differences between bacteria and fungi assessed using Student's *t*-test. Statistical significance is indicated as follows: ns, *P* ≥ 0.05; *, *P* < 0.05; **, *P* < 0.01; ***, *P* < 0.001; ****, *P* < 0.0001. Pie charts indicate the proportion of ASVs with heritability estimates (*H*^2^) exceeding 0.1, 0.3, and 0.5 thresholds within each niche. b) Boxplots showing ASV-level heritability across heritable microbial families. Individual points are jittered to show the distribution and sample size within each group.

To gain insight into the potential ecological roles of highly heritable microbes, the correlations between their relative abundance and predicted metabolic pathways were explored (Fig. S2). The analysis revealed that these bacterial groups were significantly and positively associated with multiple core metabolic pathways, including tryptophan metabolism, proline metabolism, and the S-adenosyl-L-methionine (SAM) cycle. Notably, these pathways have been extensively implicated in the regulation of plant growth, hormone biosynthesis, and adaptive stress responses. Collectively, these results suggested that highly heritable microbes may facilitate host plant adaptation by modulating rhizosphere metabolic networks, thereby optimizing nutrient acquisition and enhancing environmental stress tolerance.

### MGWAS identifies host genetic regions associated with heritable root-associated microbes

To further investigate the genetic basis underlying host–microbe interactions, MGWAS was performed at 3 taxonomic levels (family, genus, and ASV). By clustering the significant SNPs based on physical proximity, a total of 844 and 316 significant loci associated with bacteria and fungi were identified, respectively (Fig. 4a and Table S7). To explore the biological functions of these associated regions, GO and KEGG enrichment analyses on their adjacent candidate genes were performed (Table S8).

**Figure 4:**
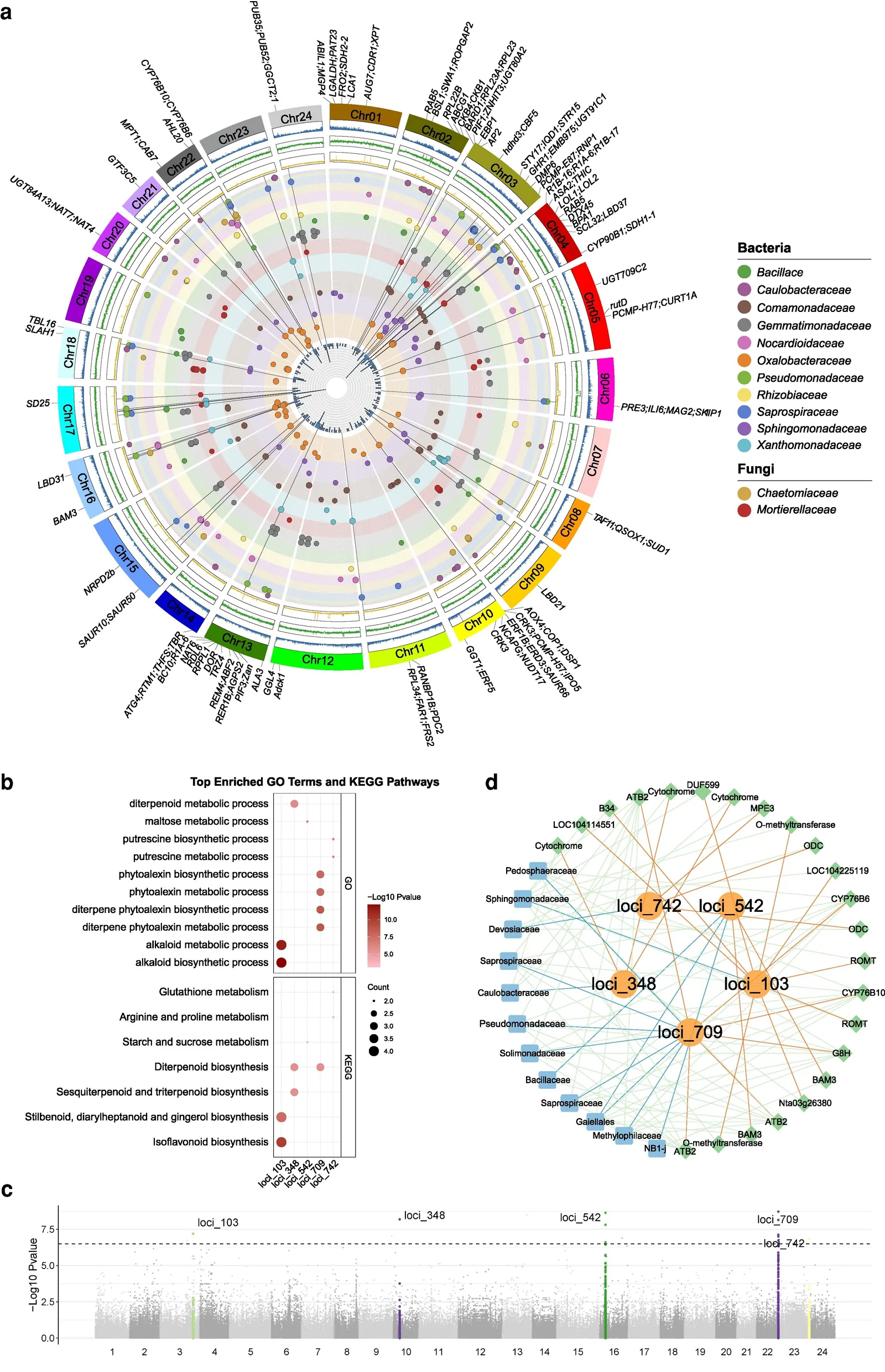
Functional characterization of host genetic loci associated with root-associated microbiota. a) Radial plot showing significant host genetic loci associated with microbial traits identified by MGWAS. The inner histogram shows the genomic density of associated loci across ∼10 Mb windows. Concentric layers represent microbial families, dots represent associated loci, larger dots indicate pleiotropic loci, and radial lines connect loci involved in the same intercategorical pleiotropic association. b) Top 10 enriched GO Biological Process terms and KEGG pathways based on genes within the corresponding locus regions. Bubble size indicates the number of annotated genes, and color indicates enrichment significance. The 10 enriched pathways were associated with 5 host genetic loci. c) Manhattan plot showing the distribution of significant MGWAS associations, with the 5 loci corresponding to the enriched pathways highlighted. d) Network diagram illustrating the relationships between MGWAS-associated loci, genes located within associated regions, and microbial taxa. Node colors and shapes denote different element types (loci, genes, microbes), and edge colors indicate distinct types of putative interactions.

For bacterial communities, 5 MGWAS-associated regions located on chromosomes 3, 10, 16, 22, and 24 were significantly enriched in secondary metabolic pathways related to plant stress responses (Fig. 4b, c). Summary statistics and model calibration for representative associations were provided in Table S9 and Fig. S3, with Q–Q plots indicating generally appropriate control of population structure and test-statistic inflation. The candidate genes in loci_103 and loci_742 were primarily involved in resistance-related secondary metabolic pathways such as alkaloid, flavonoid, and polyamine biosynthesis, whereas loci_348 and loci_709 were associated with diterpenoid biosynthesis. These findings suggested that genes located within these regions may be associated with host defense-related functions. Additionally, loci_542 was enriched in sugar metabolism and carbon source response pathways, with β-amylase 3 (*BAM3*) located within this associated region and annotated as being related to plant stress responses. Further network analysis revealed that several significant MGWAS-associated regions formed interaction modules with microbial groups and functional genes (Fig. 4d). For instance, loci_103, loci_348, and loci_742 were significantly correlated with microbial groups such as *Sphingomonadaceae*, *Caulobacteraceae*, and *Devosiaceae*, respectively. The corresponding genes located within these MGWAS-associated regions include O-methyltransferase and ornithine decarboxylase, both involved in secondary metabolism. Moreover, loci_709 showed a significant association with multiple microbial groups, including *Pseudomonadaceae*, *Saprospiraceae*, and *Methylophilaceae*. Genes located within this MGWAS-associated region included cytochrome P450 family members *CYP76B6* and *CYP76B10*, as well as the auxin-inducible protein PCNT115. These genes have annotated functions related to secondary metabolism or hormone signaling and therefore represent potential candidates for further functional investigation.

For fungal communities, the 10 most significantly enriched pathways were associated with 4 MGWAS-associated regions located on chromosomes 1, 3, 18, and 22, all of which were functionally related to plant metabolism and stress responses (Fig. S4a, b). Further analysis showed that these regions were significantly associated with the fungal family *Mortierellaceae* (Fig. S4c). For instance, the loci_276 region harbored several terpenoid biosynthesis-related genes (eg *TPS36*, *TPS2*), which were involved in the biosynthesis of sesquiterpenes and triterpenes, suggesting that genes within this region may be associated with secondary metabolite-related processes and may provide potential candidates for investigating the observed *Mortierellaceae* association. The loci_246 region contained the gene *GSTU8* (glutathione S-transferase TAU 8), which was involved in glutathione metabolism that contributed to antioxidant and toxin metabolism. The loci_1 included several ferric reduction oxidase genes, associated with iron ion transport and mitochondrial metabolism. The loci_45 region comprised genes related to RNA modification and glycosylation, whose annotated functions may be relevant to root cellular processes associated with microbial interactions. The network analysis further illustrated potential interactions between host regulatory regions, functional genes, and *Mortierellaceae* ASVs (Fig. S4c), indicating that these MGWAS-associated regions may be linked to metabolic processes potentially relevant to symbiotic or defensive interactions with this fungal group. These findings highlighted the complex interplay between host genes and microbial communities, indicating distinct functional roles among microbial groups and reflecting their diverse contributions to plant adaptation and development.

### Host genetic variation is associated with microbial assembly and metabolite variation

Host genotype might shape microbial assembly via metabolite-mediated pathways. Given that genes associated with loci_103, loci_348, and loci_709 were enriched in alkaloid and terpenoid biosynthetic pathways, mediation analysis was performed using metabolite modules as intermediate variables to evaluate whether host genotype indirectly affects microbial abundance. Representative SNPs were selected for each locus, and dominant/recessive models were applied for genotyping. Metabolite modules highly correlated with alkaloids and terpenoids were identified by WGCNA, and modules with correlation coefficients greater than 0.5 were retained (Table S10 and Fig. S5). Genotype-stratified analysis revealed that metabolite modules enriched in alkaloids (MEbrown, MEred, MEdarkred, MEmagenta) showed significantly different abundances between genotypes at loci_103, which were mainly enriched in alkaloids, particularly pyrrole and quinoline alkaloids. The representative SNP at loci_709 was significantly negatively associated with the MElightcyan and MEtan modules, with MEtan displaying a strong correlation with diterpenoids (*R*^2^ = 0.84). No significant associations were observed between loci_348 and alkaloid- or terpenoid-related metabolite modules (Fig. 5a and Table S11).

**Figure 5:**
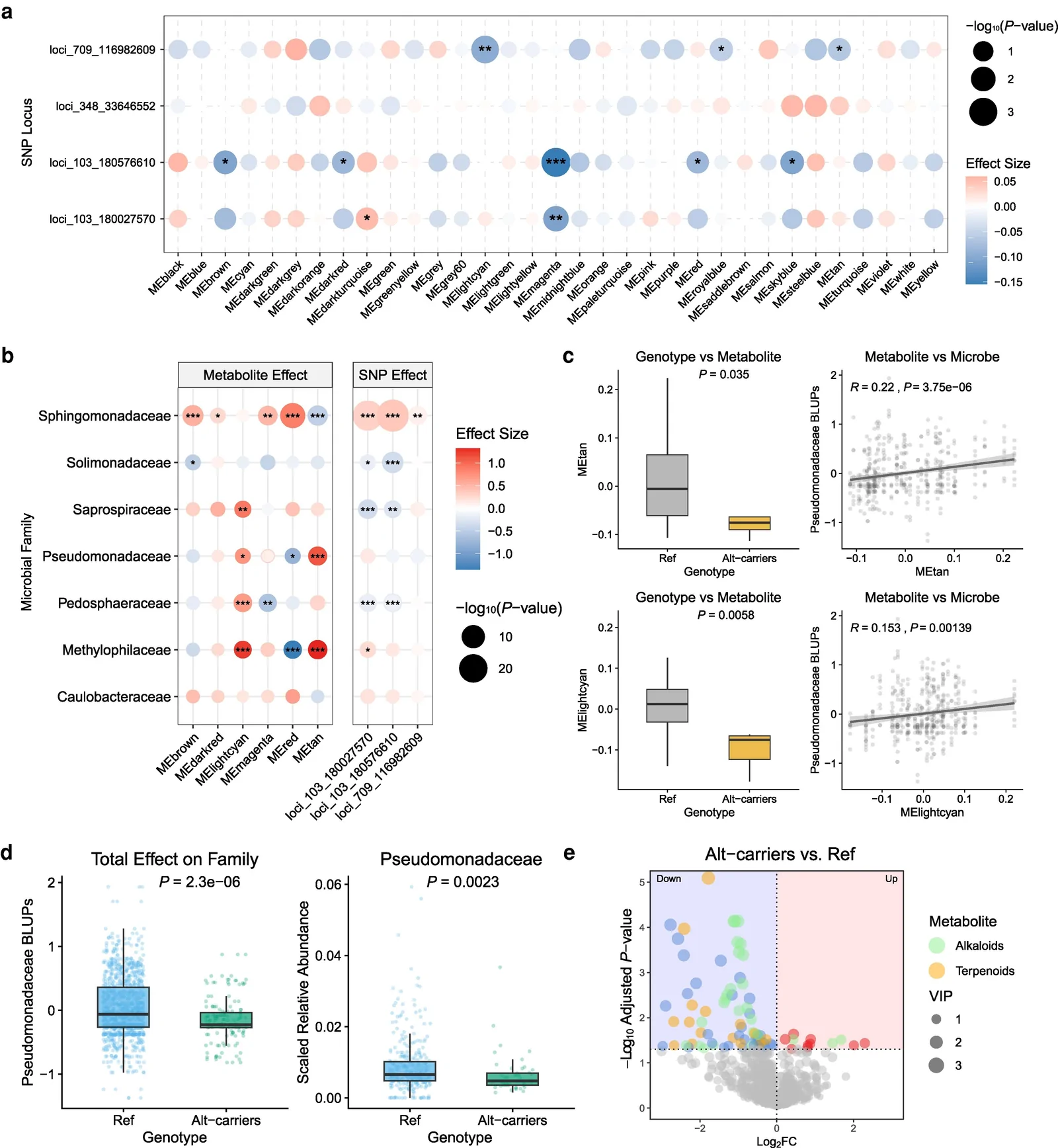
Mediation analysis of the associations among host genetic variation, metabolite variation, and microbial assembly. a) Associations between representative host genotypes (SNPs from loci_103, loci_348, and loci_709) and metabolite modules identified by WGCNA. Metabolite analyses were based on 3 independent biological replicates per cultivar. b) Associations between genotype-associated metabolite modules and microbial families, with host genotype modeled as a random effect. Bubble size indicates association strength and color represents effect size (Estimate), significance is denoted by asterisks. c) Differences in MElightcyan and MEtan module abundances among loci_709 genotypes and their correlations with *Pseudomonadaceae* BLUP values. d) Genotype-dependent differences in *Pseudomonadaceae* BLUP values and relative abundance at loci_709. Statistical significance was evaluated using Wilcoxon rank-sum tests. e) Volcano plot of differentially accumulated metabolites between mutation-containing and reference genotypes at loci_709.

Further association analysis incorporating microbial BLUP values and host genotype as a random effect revealed distinct association patterns among microbial families (Fig. 5b and Table S12). *Caulobacteraceae* showed no significant association with metabolite modules or genotype. In contrast, *Pseudomonadaceae* was significantly associated only with terpenoid-related modules, whereas *Sphingomonadaceae*, *Pedosphaeraceae*, and *Methylophilaceae* exhibited significant associations with both genotype and metabolite modules. Consistently, the terpenoid-related modules MElightcyan and MEtan differed significantly among loci_709 genotypes and were positively correlated with *Pseudomonadaceae* abundance (Fig. 5c), and the relative abundance of *Pseudomonadaceae* was significantly lower in cultivars carrying loci_709 mutations (Fig. 5d). Among the genotype–metabolite–microbe associations, the loci_709–MEtan–*Pseudomonadaceae* pathway showed a significant indirect effect, with a bootstrap 95% CI excluding zero and approximately 37% of the total effect mediated through MEtan (Fig. S6 and Table S4). Differential metabolite analysis further showed that, compared with the reference genotype, mutation-containing genotypes exhibited predominantly downregulated metabolites (Down: 74; Up: 12), with alkaloids (25, 34%) and terpenoids (15, 20%) accounting for the majority of changes (Fig. 5e and Table S13).

Collectively, these results supported an association between host genetic variation, secondary metabolite variation, and root-associated microbial abundance, with partial mediation observed for the loci_709–MEtan–*Pseudomonadaceae* pathway.

### Genetically inherited root-associated microbes contribute to host disease resistance

To translate genotype-associated microbial taxa into functionally testable isolates, high-throughput cultivation of mixed root-associated samples was performed. In total, 546 bacterial and 72 fungal strains were isolated, and their sequences were matched against the amplicon sequencing dataset at ≥95% sequence identity, resulting in matches to 735 bacterial and 41 fungal ASVs (Fig. 6a and Fig. S7). To prioritize candidates for functional validation, we focused on microbial taxa that showed strong host genetic associations in MGWAS analyses, whose associated host loci were enriched in secondary metabolite and defense-related pathways, and that exhibited relatively high heritability estimates. This genotype-guided strategy leveraged natural host genetic variation and microbial recruitment patterns observed under field conditions, thereby enriching for microbial taxa with a higher likelihood of contributing to host phenotypes.

**Figure 6:**
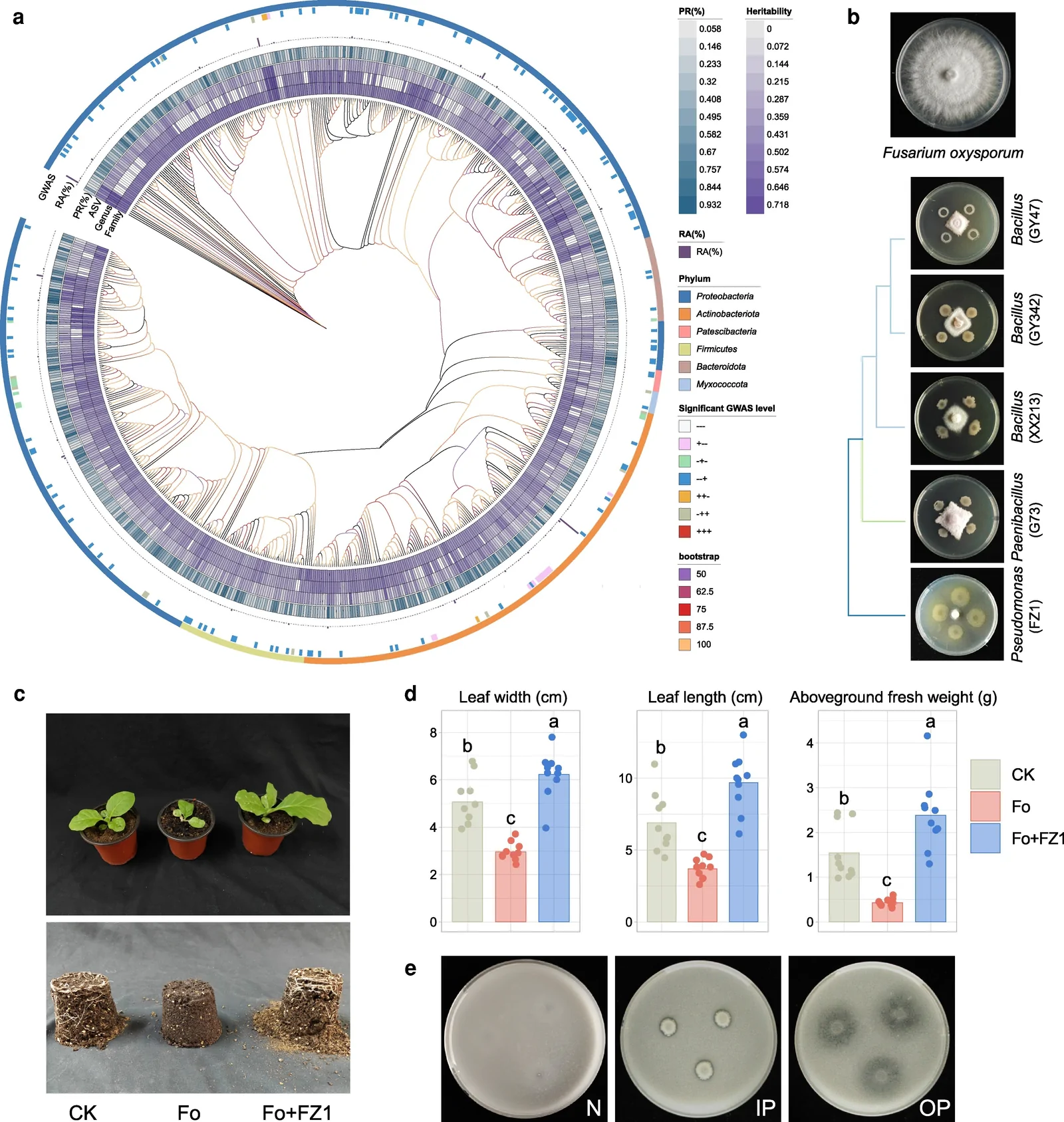
Evaluation of the antagonistic and growth-promoting potential of rhizosphere bacterial strains against *F. oxysporum*. a) Phylogenetic and host-association overview of culturable bacteria isolated from tobacco root-associated samples. The tree summarizes taxonomic relationships, host-association significance, heritability estimates across taxonomic levels, and ASV prevalence. (b) In vitro dual-culture assay showing the inhibitory effects of 5 antagonistic bacterial strains on the mycelial growth of *F. oxysporum* on PDA medium. c) Phenotypic performance of tobacco plants 7 d posttreatment with: no pathogen (negative control) (CK), *F. oxysporum* only (positive control) (Fo), and *F. oxysporum* + strain FZ1 (Fo + FZ1). Both shoot and root development are shown (*n* = 10 independent biological replicates per treatment). d) Quantification of tobacco growth parameters following inoculation with strain FZ1. Different lowercase letters indicate significant differences among treatments based on *Duncan*'s multiple range test (*P* < 0.05). (e) Growth performance of strain FZ1 on nitrogen-free (N), organic phosphate (OP), and inorganic phosphate (IP) media, indicating potential plant growth-promoting traits.

Several cultivable bacterial taxa meeting these criteria belonged to genotype-associated families identified in the MGWAS analysis. To evaluate whether such genetically inherited microbes contribute to disease resistance, we selected tobacco root rot caused by *F. oxysporum* as a representative pathosystem. Antagonistic assays identified 5 bacterial isolates with significant inhibitory activity against *F. oxysporum*, including 3 *Bacillus* strains (GY47, GY342, and XX213), one *Paenibacillus polymyxa* strain (G73), and one *Pseudomonas aeruginosa* strain (FZ1), corresponding to 45 ASVs in the community dataset (identity >95%) (Fig. 6b and Table S14). Among them, FZ1 exhibited the strongest antagonistic activity and nearly completely suppressed fungal growth, whereas the remaining strains showed moderate but significant inhibition. Correlation analyses further revealed that ASVs corresponding to these antagonistic bacteria were positively associated with multiple antimicrobial secondary metabolites, including pyridine alkaloids, free fatty acids, organic acids, and terpenoids (*P* < 0.05, |cor| > 0.3) (Fig. S8). These metabolites are frequently implicated in plant defense and pathogen suppression, suggesting that genetically inherited antagonistic bacteria may contribute to disease resistance through interactions with rhizosphere metabolic processes.

To assess their biocontrol efficacy in planta, pathogenic inoculation experiments were conducted under soil cultivation conditions. Seven days after *F. oxysporum* inoculation, plants in the pathogen-only (Fo) and Fo plus *E. coli* (Fo + Ec) treatments exhibited severe disease symptoms, including growth inhibition, leaf wilting, and chlorosis (Fig. S9a). Consistently, leaf length, leaf width, and shoot fresh weight were significantly reduced relative to control plants (*P* < 0.05) (Fig. S9b). In contrast, inoculation with antagonistic bacteria substantially alleviated disease symptoms. Plants treated with XX213 displayed growth comparable to controls, whereas FZ1 and GY47 also significantly improved plant performance relative to the pathogen-only treatment (*P* < 0.05), accompanied by enhanced root development and reduced symptom severity (Fig. 6c, d and Fig. S9a, b). Functional assays further demonstrated that GY47, XX213, and G73 possessed potassium-solubilizing activity, whereas FZ1 showed strong phosphate-solubilizing capacity, particularly for organic phosphorus, together with weak nitrogen-fixing ability (Fig. 6e and Fig. S9c). These traits are consistent with improved nutrient mobilization and plant nutrient acquisition.

Collectively, these results suggest that host genotype-associated root bacteria can enhance plant performance through both pathogen suppression and nutrient mobilization. Notably, FZ1 exhibited the strongest antagonistic activity and showed close associations with defense-related metabolites. Combined with the mediation analysis indicating that *Pseudomonadaceae* recruitment was strongly linked to host genotype through terpenoid-associated metabolic modules, these findings highlighted FZ1 as a representative model for further investigation of host–microbe interaction mechanisms.

### FZ1 elevated tobacco resistance in association with defense activation and rhizosphere homeostasis

To elucidate the antagonistic mechanisms of FZ1 against *F. oxysporum*, we first performed whole-genome sequencing and functional annotation for its genome. The FZ1 genome was approximately 6.28 Mb in size with a GC content of 66.58%, encoding 5,737 predicted protein-coding genes. Comparative genomic analysis showed the highest similarity to *P. aeruginosa* (Fig. S10 and Fig. S11a). Functional annotation revealed extensive genetic potential related to antifungal activity and rhizosphere adaptation, including gene clusters involved in the biosynthesis of phenazine, furanomycin, and multiple siderophores, as well as genes associated with ABC transporters, quorum sensing, and biofilm formation. Additionally, genes related to nitrogen metabolism and carbohydrate transport were identified, implying a potential role of FZ1 in facilitating plant nutrient uptake (Fig. S11b). Secondary metabolite analysis predicted 16 BGCs encompassing NRPS, RiPPs, phenazine, homoserine lactone, thiopeptide, and polyketide pathways. Notably, pyoverdine, pyochelin, and pseudopaline, 3 siderophores typically secreted by *P. aeruginosa*, were also observed, which may mediate antifungal activity (Fig. S11c) (Ghssein and Ezzeddine 2022).

To assess whether FZ1 was associated with tobacco resistance through modulation of host defense responses, transcriptome profiling of root tissues was conducted at 7 d postinoculation. Compared with the control, plants infected with *F. oxysporum* (Fo) exhibited 339 upregulated and 361 downregulated genes. In contrast, co-inoculation with FZ1 (Fo + FZ1) induced a markedly stronger transcriptional response, with 1,031 genes upregulated and 262 downregulated, indicating substantial host transcriptional reprogramming triggered by FZ1 (Fig. 7a). GO enrichment analysis showed that although *F. oxysporum* activated certain immune-related pathways, it concomitantly suppressed critical defense responses including chitin recognition, protein kinase activity, and reactive oxygen species signaling (Fig. 7b). This suppression may involve proteases and effectors that interfere with chitin-triggered immunity and pathogen-associated molecular patterns recognition, potentially contributing to reduced host resistance (Tintor et al. 2020). Strikingly, FZ1 co-inoculation counteracted this pathogen-mediated suppression by robustly activating defense-related pathways associated with chitin response, hormone signaling, and secondary metabolite biosynthesis, particularly terpenoids, sulfur-containing compounds, and phytoalexins. Key regulatory components such as MAPK cascade signaling, the chitin receptor *LYK4*, calcium signaling, and terpene synthases were specifically upregulated in the Fo + FZ1 group, with no significant induction observed in either the Fo or control groups (Fig. S12). Collectively, these findings suggest that FZ1 restores plant immunity by inducing core defense pathways that are subverted during *F. oxysporum* infection.

**Figure 7:**
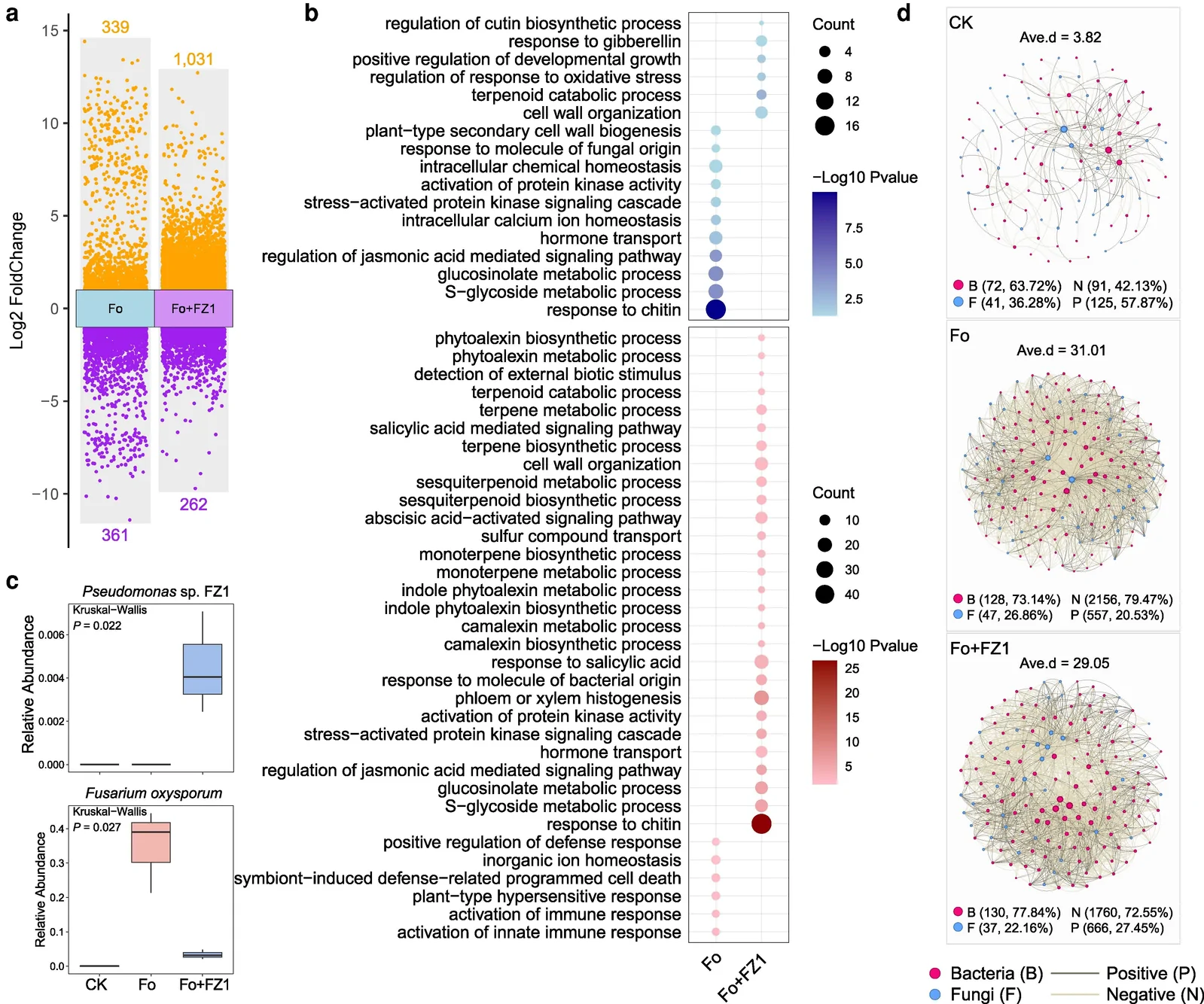
Effects of *P. aeruginosa* FZ1 on root transcriptome profiles and rhizosphere microbial community stability in tobacco. a) Number of differentially expressed genes (DEGs) in tobacco roots in response to *F. oxysporum* (Fo) or Fo combined with FZ1 (Fo + FZ1), relative to the control group (*n* = 3 independent biological replicates per treatment). b) GO Biological Process enrichment analysis of significantly upregulated (red) and downregulated (blue) genes, visualized as a bubble plot indicating the number of annotated genes (bubble size) and enrichment significance (color). c) Relative abundance of FZ1 and *F. oxysporum* in different treatment groups. d) Cross-domain co-occurrence network illustrating microbial community interactions in the rhizosphere under different treatments.

To further assess the ecological consequences of FZ1 colonization, rhizosphere microbial community dynamics were characterized after 7 d of greenhouse cultivation. Amplicon sequencing analysis revealed comparable bacterial alpha-diversity across treatments, whereas fungal diversity progressively declined from CK to Fo to Fo + FZ1 groups (Fig. S13a–d). Consistent with disease suppression, the relative abundance of *F. oxysporum* was markedly elevated in the Fo group but significantly reduced upon FZ1 co-inoculation, confirming successful rhizosphere colonization and pathogen suppression by FZ1 (Fig. 7c). Microbial co-occurrence network analysis revealed that the networks in Fo and Fo + FZ1 groups exhibited higher network connectivity, with increased proportions of negative correlations, particularly between bacterial-bacterial and bacterial-fungal pairs, indicating intensified interspecific competition (Fig. 7d and Fig. S13e, f). Notably, Fo treatment produced a more fragile yet tightly clustered network structure, whereas FZ1 co-inoculation partially alleviated this effect and enhanced network stability. Together, these results demonstrate that FZ1 enhances tobacco resistance through both direct and indirect mechanisms, including suppression of *F. oxysporum* in the rhizosphere and activation of host defense responses.

## Discussion

Plants and their associated microorganisms form co-evolved functional units that enhance host adaptation to environmental challenges (Trivedi et al. 2020). However, modern breeding may inadvertently disrupt genotype–microbiome interactions and thereby limit the recruitment of beneficial microbes (de Vries et al. 2020). Understanding how host genetic variation shapes microbiome assembly is therefore essential for microbiome-informed crop improvement (Ge and Wang 2025). In this study, by integrating population genomics, microbiome profiling, metabolomics, and functional validation, we identified genotype-associated microbial taxa and revealed a metabolite-mediated link between host genetic variation, microbiome recruitment, and disease resistance. These findings provide insight into the genetic basis of plant–microbiome interactions and support the development of microbiome-informed breeding strategies.

Although soil environmental factors remain the primary drivers of microbial community structure, host genotype exerts a substantial and nonnegligible influence, particularly in the rhizosphere and rhizoplane compartments, where it accounted for approximately 30% of the explained variation. Similar observations have been reported in maize (Meier et al. 2022), millet (Wang et al. 2022b), and switchgrass (Edwards et al. 2023), indicating that host genetic effects on root-associated microbiomes are a conserved feature across crop species. Notably, host genetic effects increased from the endosphere to the rhizosphere, suggesting that genetically associated microbial recruitment is strongest at the root–soil interface. The endosphere imposes stringent physical and immune barriers on microbial colonization, whereas the rhizosphere provides a chemically dynamic environment in which microorganisms respond to host-derived metabolites, signaling molecules, and nutrient fluxes (van der Heijden and Schlaeppi 2015; Wagner 2021). Together, these observations identify the rhizosphere as a major arena for genetically mediated host–microbiome interactions.

At the taxonomic level, several bacterial families, including *Burkholderiaceae*, *Xanthomonadaceae*, *Bacillaceae*, *Pseudomonadaceae*, and *Rhizobiaceae*, were enriched among highly heritable ASVs. Comparable patterns have been reported in millet (Wang et al. 2022b), sorghum (Deng et al. 2021), maize (He et al. 2024), and tomato (Oyserman et al. 2022), suggesting that plants may preferentially exert genetic control over microbial groups with growth-promoting or stress-mitigating functions (Trivedi et al. 2020; Pereira et al. 2023). Nevertheless, microbial heritability is context dependent and can vary across environmental conditions, highlighting the importance of genotype-by-environment interactions in shaping microbiome inheritance.

Our MGWAS analysis identified numerous host loci associated with microbial taxa; however, most significant variants resided in noncoding genomic regions, complicating direct functional interpretation. To overcome this limitation, we integrated GO enrichment analyses of genes surrounding significant SNPs and synthesized associations across multiple microbial taxa to infer putative functional linkages. This strategy revealed host genetic regions enriched in secondary metabolite biosynthesis pathways and guided the isolation of functionally relevant microorganisms, including 5 bacterial strains antagonistic to *F. oxysporum*. Among these, *P. aeruginosa* FZ1 emerged as a representative genotype-associated microbe with strong disease-suppressive activity.

Mediation analysis indicated that the genetic effect of loci_709 on *Pseudomonadaceae* abundance was partly transmitted through the terpenoid-associated metabolite module MEtan, with a significant indirect effect (bootstrap 95% CI) and proportion mediated of approximately 37%. These results support a partial, metabolite-linked mediation rather than a predominantly mediated pathway. Terpenoids are well-established regulators of rhizosphere microbial assembly, functioning both as antimicrobial compounds and ecological signals that shape microbial community composition (Lv et al. 2024). In *Solanaceae* species, diterpenoids such as casbene, phytuberin, and solavetivone have been reported to selectively enrich terpene-adapted microorganisms while simultaneously contributing to plant defense responses (Fiesel et al. 2022). Consistent with this mechanism, *Pseudomonas* species harbor diverse terpene catabolic pathways that enable the utilization and transformation of plant-derived terpenoids (Yu et al. 2026). The strong association between the diterpenoid-enriched module MEtan and *Pseudomonadaceae* abundance, together with the enrichment of terpene biosynthetic genes (eg *CYP76B6* and *CYP76B10*) near significant host loci, is consistent with a potential link between host genetic variation, rhizosphere terpenoid composition, and *Pseudomonadaceae* abundance (Figs 4 and 5).

Beyond microbial recruitment, plant terpenoids may also contribute directly to pathogen suppression. Previous studies have demonstrated antifungal activities of diverse terpenoid compounds against *Fusarium* species, suggesting that genotype-dependent variation in rhizosphere terpenoid composition could influence disease outcomes through both chemical and microbiological mechanisms (Lin et al. 2025). The enrichment of terpenoid biosynthesis genes near MGWAS-associated loci is consistent with a potential connection between host genetic variation, terpenoid metabolism, and disease-associated microbial traits. Together, these findings provide a framework linking host genetic variation with secondary metabolism, root-associated microbiome composition, and disease resistance, while suggesting potential biological connections among these processes.

In contrast, other heritable microbial families, including *Methylophilaceae* and *Sphingomonadaceae*, exhibited only partial mediation effects, indicating that metabolite variation alone cannot fully explain the association between host genetic variation and microbial abundance (Fig. 5). These observations suggest that different microbial groups may be associated with host genetic variation through distinct biological processes. In addition to metabolic cues, genotype-dependent immune signaling, rhizosphere physicochemical properties, and microbe–microbe interactions may also contribute to microbiome assembly. Collectively, our results support a multilayered framework linking host genetic variation, metabolite variation, and root-associated microbial communities.

Regarding potential applications, the use of *P. aeruginosa* FZ1 as an agricultural biocontrol agent warrants caution. Although FZ1 exhibited strong antifungal activity in vitro and was associated with reduced disease severity in greenhouse trials, *P. aeruginosa* was a well-documented opportunistic human pathogen, and environmental release of this species raised biosafety and regulatory concerns (Letizia et al. 2025). Its deployment would require strain-specific risk assessment, appropriate containment, and compliance with relevant national and international regulations. We therefore regard FZ1 primarily as a model strain for studying host–microbe interactions rather than as an immediately deployable biocontrol agent.

Despite these insights, several limitations should be acknowledged. The relatively modest population size (50 cultivars) may have reduced the statistical power to detect host genetic effects on microbiome variation. In addition, the MGWAS-associated intervals may contain multiple genes, and the prioritization of individual genes in this study was primarily based on their genomic proximity to significant markers and their annotated functions. Therefore, these genes should be considered potential candidates rather than functionally validated causal genes, and further genetic and functional characterization will be required to determine their specific contributions to host–microbiome associations. Furthermore, disease resistance phenotypes were not systematically evaluated across the entire cultivar panel, limiting direct integration of host resistance traits into the MGWAS framework. In addition, amplicon sequencing provides limited functional resolution compared with metagenomic and metatranscriptomic approaches. Future studies incorporating larger populations, multi-environment validation, pathogen challenge experiments, and functional genomics will be essential for establishing causal links among host genotype, metabolite production, microbiome assembly, and disease resistance. Nevertheless, our findings provide a framework linking host genetic variation, secondary metabolism, microbiome composition, and disease resistance, and highlight potential directions for hologenome breeding and host-guided microbiome engineering.

## Supplementary Material

kiag727_Supplementary_Data

## Data Availability

The raw sequencing data generated in this study, including 16S/ITS amplicon sequencing data, whole-genome resequencing data of the tobacco population, and rhizosphere metabolomic profiles, were deposited in the Genome Sequence Archive under the BioProject accession number PRJCA041117. Additional processed data and analysis scripts are available from the corresponding author upon reasonable request.
