# Supplementary material for: Host genetic variation is associated with root-associated microbiome assembly and resistance to Fusarium oxysporum in tobacco
Source: Plant Physiol. 2026 Sep 30;202(2):kiag727. doi: 10.1093/plphys/kiag727 (PMC13648874; doi:10.1093/plphys/kiag727)
Supplement: Supplementary file 1 [file kiag727_supplementary_data.zip › Supplementary figures_revised.docx]

**Host Genetic Variation Is Associated with Root-Associated Microbiome Assembly and Resistance to *Fusarium oxysporum* in Tobacco**

**Fig. S1** **Genetic variation in the tobacco population.** (A) Sampling map indicating geographic locations of the accessions. (B) Comparison of variant detection results across different software tools. (C) Density distribution of SNPs across chromosomes. (D) Bar plot showing the number of different types of genetic variants. (E) Maximum likelihood phylogenetic tree constructed using SNP data. Agronomic traits of each variety are annotated on the right, as indicated in the legend. (F) Population structure analysis based on the Admixture model. The lowest cross-validation (CV) error (0.436) was observed at *K* = 3.

**Fig. S2** **Associations between dominant bacterial taxa and functional potentials in the rhizosphere.** Heatmap showing significant *Spearman’s* correlations between the relative abundances of dominant bacterial families and predicted KEGG metabolic pathway abundances (inferred via PICRUSt2) in rhizosphere (RS) samples. Only correlations with *P* < 0.05 and |correlation coefficient| > 0.5 are shown.

**Fig. S3. Q–Q plots assessing the calibration of MGWAS associations for major host genetic loci and microbial traits.** Q–Q plots compare the observed and expected distributions of association P values for the indicated microbial traits. Each panel represents a microbial trait–niche combination, with the corresponding major associated host genetic locus indicated in the panel title. The diagonal dashed line indicates the expected distribution under the null hypothesis. Genomic inflation factors (λ_GC_) are provided in Supplementary Table S9. MGWAS was performed using a linear mixed model incorporating the first 10 genomic principal components and a genome-wide kinship matrix to account for population structure and relatedness.

**Fig. S4** **Functional characterization of host genetic loci associated with root-associated fungi.** Figure legend is the same as that of Fig. 4B-D.

**Fig. S5** Heatmap of correlations between alkaloid/terpenoid metabolites and WGCNA modules

**Fig. S6 Mediation analysis of host genotype–metabolite–microbe pathways.** (A) Indirect effect (*a*×*b*) with bootstrap 95% confidence intervals (1,000 resamples) for all 66 tested pathways (overview), filled colored points denote pathways whose 95% CI excludes zero (red, significant negative; green, significant positive), grey points denote non-significant pathways, and triangles denote suppression effects (indirect and direct effects of opposite sign). (B) Enlarged view of the eight *Pseudomonadaceae* pathways, MEtan is the module with a significant indirect effect consistent with partial mediation.

**Fig. S7** **Characterization of culturable fungi from the tobacco root-associated samples.** The phylogenetic tree is colored according to bootstrap values, as indicated in the legend. The inner purple rings represent the broad-sense heritability estimates at the family, genus, and ASV levels, while the blue ring denotes prevalence. Bar plots display the relative abundances of each ASV. The outer rings indicate the significance of host associations at the family, genus, and ASV levels, and the corresponding phylum classification, as shown in the legend.

**Fig. S8** Heatmap showing the number and types of rhizosphere metabolites that are positively correlated with ASVs associated with antagonistic bacteria.

**Fig. S9** **Evaluation of the antagonistic and growth-promoting potential of heritable bacterial strains against *F. oxysporum*.** (A) Phenotypic performance of tobacco seedlings 7 days after treatment: control without pathogen (left), pathogen + blank medium (middle), and pathogen + bacterial strains (right). Both shoot and root development are shown. (B) Quantification of tobacco growth parameters following inoculation with each strain (n = 10 independent biological replicates per treatment). Different lowercase letters indicate significant differences among treatments based on *Duncan’s* multiple range test (*P* < 0.05). (C) Growth performance of antagonistic strains on Alexandrov medium, reflecting their potassium-solubilizing ability, as indicated in the legend.

**Fig. S10** **Summary of taxonomic and functional annotations of *P. aeruginosa* FZ1.** Bar plots showing the number of annotated species and genes.

**Fig. S11 Genomic overview and gene function annotations of *P. aeruginosa* FZ1 genome.** (A) Circular genome map of strain FZ1. (B) Number of genes annotated in KEGG pathways related to antifungal activity. (C) Predicted secondary metabolite biosynthetic gene clusters identified by antiSMASH.

**Fig. S12** **Heatmap of defense-related and secondary metabolism-related gene expression after inoculation with *F. oxysporum* and/or antagonistic strain FZ1.** Gene expression values are shown as TPM and normalized by column (z-score).

**Fig. S13** **Effects of *P. aeruginosa* FZ1 on rhizosphere microbial community structure.** (A-D) Alpha diversity of bacterial (A, C) and fungal (B, D) communities in different treatment groups. (E) Distribution of inter- and intra-kingdom co-occurrence relationships. (F-G) Network stability metrics across groups, showing network vulnerability (F) and cohesion (G).
